# Environmental and host- and parasite-associated drivers of the flea-mammal interaction network structure differ between biogeographic realms

**DOI:** 10.1007/s00436-025-08603-z

**Published:** 2025-11-24

**Authors:** Boris R. Krasnov, Irina S. Khokhlova

**Affiliations:** 1 Mitrani Department of Desert Ecology, Swiss Institute for Dryland Environmental and Energy Research, Jacob Blaustein Institutes for Desert Research, Ben-Gurion University of the Negev, Sede Boqer Campus, 84990 Midreshet Ben-Gurion, Israel; 2 French Associates Institute for Agriculture and Biotechnology of Drylands, Jacob Blaustein Institutes for Desert Research, Ben-Gurion University of the Negev, Sede Boqer Campus, Midreshet Ben-Gurion, Israel

**Keywords:** Climatic factors, Fleas, Functional diversity, Mammals phylogenetic diversity, Species richness

## Abstract

We evaluated the effects of climatic factors, host and parasite species richness, and their phylogenetic and functional diversity on the nestedness and modularity of mammal-flea interaction networks from four biogeographic realms. We also tested for the associations between pure structural network dissimilarity (i.e., dissimilarity between host-sharing-by-fleas networks, *Dh*, or dissimilarity between flea-sharing-by-hosts networks, *Df*) and environmental dissimilarity, and host and flea compositional, phylogenetic, and functional dissimilarities. We asked whether (a) the relative effects of these factors differ between biogeographic realms and (b) the network structure is more strongly driven by environmental, geographic, host-associated, or flea-associated factors. The climatic drivers of nestedness and modularity differed between realms. The networks in different realms responded to different factors, with the directions of some of these effects being opposite. Among interactor-associated factors, host species richness was most often detected as an important driver of nestedness, whereas flea species richness mainly affected modularity. *Dh* was mostly explained by host-associated and, to a lesser extent, flea-associated dissimilarity. No effect of host-associated dissimilarity on *Df* was detected, but it was linked with flea-associated dissimilarity in two of the four realms. Environmental dissimilarity weakly affected *Dh* and did not affect *Df.* We conclude that between-realm differences in the drivers of network structure resulted from an interplay of ecological and historical factors, whereas between-interactor differences in their effects on the network structure arose due to the asymmetry in host-flea relationships.

## Introduction

Environmental factors driving variation in species richness and composition of biological communities have been repeatedly investigated in various taxa, environments, and geographic regions (e.g., Whittaker 1967; Nakanishi et al. 2014; Geburzi et al. 2022). Recently, it has been recognized that communities are not isolated from one another; instead, species within or between communities are involved in various types of interactions, representing ecological interaction networks. Consequently, the mechanisms behind the environmental variation of communities cannot be fully elucidated without considering species interactions in these communities (see Tylianakis and Morris 2017; Pellissier et al. 2018 for reviews). This understanding resulted in a burst of studies that compared various structural metrics of ecological networks along environmental and/or geographic gradients (Schleuning et al. 2012; Kissling and Schleuning 2015; Dalsgaard et al. 2017; Poisot et al. 2017; Dallas and Poisot 2018; d’Bastiani et al. 2020; Corro et al. 2022; de Angeli Dutra and Poulin 2024; Zhang et al. 2025). In particular, it appears that, in bipartite (e.g., predator–prey, pollinator-plant, or host-parasite) networks, species richness and composition of the interactor communities, on the one hand, and the patterns of interactions, on the other hand, could respond differently to the same environmental factors (Poisot et al. 2017). This is because species interactions depend on various factors so that two species often interact under some environmental conditions but do not interact under other conditions, even if they occur in both (e.g., CaraDonna et al. 2017). In addition, evolutionary history of interactors may affect the patterns of their interactions (e.g., Peralta 2016). Given that evolution and historical dispersal of the same animal and plan taxa differ between large geographic areas such as biogeographic realms (e.g., Gibert et al. 2021 for fleas and small mammals), structural drivers of the same interaction networks may thus vary across these areas (Harmon et al. 2019).

Among the structural patterns of ecological networks, nestedness and modularity are most often studied. The nestedness of a bipartite network (i.e., consumers and resources) reflects the degree to which specialist consumers interact with a subset of the resource species used by increasingly more generalist consumers (Bascompte et al. 2003). Modularity represents the degree of network subdivision into subsets of frequently interacting species (Newman 2006; Bascompte and Jordano 2013). A module is defined as a certain group of, for example, consumer species interacting more frequently with a certain group of resource species but less so with species from another module (Olesen et al. 2007). Higher values of both metrics indicate a higher level of network stability (Bascompte 2007; Grilli et al. 2016; Dormann et al. 2017). Nestedness and modularity are not mutually exclusive structural patterns, and the same network can be simultaneously nested and modular (Fortuna et al. 2010; Pinheiro et al. 2019; Felix et al. 2022).

Investigations of the effects of environmental and/or geographic factors on nestedness and modularity have demonstrated that the patterns of their variation, along environmental or geographic (e.g., latitude) gradients, differ between different networks. For example, no consistent latitudinal pattern in insect-parasitoid networks was reported by Morris et al. (2014). Poulin and McDougall (2022) found that the modularity of fish-parasite networks decreased with increasing latitude, whereas the opposite was reported for bat-batfly networks (Biz et al. 2023). In plant-pollinator networks, modularity decreased with absolute latitude (Trøjelsgaard and Olesen 2013), but the opposite trend was found in seed-dispersal networks (Schleuning et al. 2014). Furthermore, given that the structure of the networks is, to some extent, influenced not only by local but also by regional and historical processes, such as dispersal and speciation (Hubbell 2001), geographic variation in the network structure’s response to environmental gradients can be expected (Krasnov et al. 2023). However, to the best of our knowledge, this has never been specifically studied.

Species interactions are influenced by multiple factors, including species abundances, spatial distribution, phenology, and trait complementarity (Jordano et al. 2003; Stang et al. 2006; Vázquez et al. 2009). These factors, to a great extent, are determined by the evolutionary histories of the interacting species. Consequently, the structure of interaction networks, including nestedness and modularity, should be affected not only by the interactors’ species richness (Olesen et al. 2007) but also by their phylogenetic and/or functional diversity (Chamberlain et al. 2014; Luis et al. 2015; Watts et al. 2016; Mello et al. 2019; Higino and Poisot 2021). It is, however, unclear whether the effects of these interactor-associated factors on the network structure are stronger or weaker than the effects of environmental or geographic factors.

Variation in the network structure along environmental gradients may also be quantified by relating structural dissimilarity between networks to environmental differences (Pellissier et al. 2018). There are two main classes of network dissimilarity metrics. In one class, dissimilarity between networks considers both species identities and species interactions (Poisot et al. 2012). This, however, may mask pure structural differences (Dallas and Poisot 2018). Another class of network dissimilarity metrics is based on graph theory (Schieber et al. 2017; Tantardini et al. 2019). These metrics aim to estimate only dissimilarity in the network topological structure, without taking into account interactor identities. One of these metrics, the D statistic (Schieber et al. 2017), quantifies network dissimilarity from the purely structural perspective.

Here, we investigated the environmental, geographic, and interactor-associated drivers of the structure of small mammal-flea networks from four biogeographic realms (Afrotropical, Nearctic, Neotropical, and Palearctic) and asked whether (a) the relative effects of these factors differ between biogeographic realms and (b) the network structure is more strongly driven by environmental, host-associated, or flea-associated factors. This was carried out in two ways, namely one that focused on the α- (nestedness and modularity) and one that focused on the β-properties of the networks (sensu Pellissier et al. 2018). In the analyses of the α-properties, we evaluated the effects of (a) environment (air temperature, precipitation, and climatic seasonality), (b) geography (absolute latitude), and (c) host and flea species richness, phylogenetic diversity, and functional diversity on network nestedness and modularity. In the analyses of the β-properties, we tested for the associations between the networks’ pure structural dissimilarity quantified using D statistics (see below) and (a) environmental dissimilarity, (b) host and flea compositional dissimilarity, (c) host and flea phylogenetic dissimilarity, and (d) host and flea functional dissimilarity. We used air temperature, precipitation, and seasonality as environmental predictors because these factors have been shown to affect nestedness and modularity in the interaction networks (e.g., Schleuning et al. 2012; Corro et al. 2022; but see Brimacombe et al. 2022). The effects of environmental, as well as interactor-associated, dissimilarity on pure structural between-network dissimilarity have never been specifically studied (except geographic distance; see Krasnov et al. 2024a). Characterization of the network dissimilarity from purely structural perspective (that is, considering interactions only without taking into account species identities) may reveal patterns obscured when the traditional methodology is used (Dallas and Poisot 2018; Krasnov et al. 2024a).

## Materials and methods

### Data on flea distribution

The data on interactions between fleas and small (< 5 kg; Degen 1997) mammals (Didelphimorphia, Paucituberculata, Macroscelidea, Eulypotyphla, Rodentia, and ochotonid Lagomorpha) were taken from our earlier studies (e.g., Krasnov et al. 2022a). These data were obtained from literature sources for 15 regions of the Afrotropics (207 flea and 196 host species), 23 regions of the Nearctic (258 flea and 221 host species), 17 regions of the Neotropics (196 flea and 258 host species), and 36 regions of the Palearctic (326 flea and 215 host species) (see the list of regions, maps, and references in Krasnov et al. 2022a). We relied on the information in the original sources and did not consider interactions identified as accidental or occasional by the authors of the sources.

### Environmental variables and latitude

Each region was characterized by seven climatic variables (air temperature and precipitation of the warmest quarter, air temperature and precipitation of the coldest quarter, annual precipitation, and air temperature and precipitation seasonality) and one environmental variable (net primary production). Temperature seasonality was calculated as the standard deviation of mean monthly temperatures, whereas precipitation seasonality was calculated as coefficient of variation of monthly precipitation totals (Fick and Hijmans 2017). Data on these variables were obtained from the CHELSA 2.1 dataset (Karger et al. 2018; Brun et al. 2022). For each region, these data were taken for a 1000-km buffer zone around the region’s geographic center and averaged across cells of 30 arc seconds (~ 1 km^2^). The raw values of the climatic variables were then substituted with the scores of the first principal component extracted for each category of environmental variables [air temperature (T), precipitation (P), and seasonality (S)]. The values of T and P correlated positively with the respective original variables and explained 63%–98% of their variation. The values of S correlated positively with both temperature and precipitation seasonality in the biogeographic realms of the Old World (the Afrotropics and the Palearctic), explaining 51–54% of the variation. However, in the biogeographic realms of the New World (the Neotropics and the Nearctic), S correlated positively with precipitation seasonality, but negatively with temperature seasonality and explained 55–68% of the variation. Latitudinal values of the regions’ geographic centers were obtained using ArcGIS 10.6.

### Host and flea phylogenies

Phylogenetic trees for hosts and fleas were separately constructed for each biogeographic realm. Phylogenetic trees for hosts were taken as 1000 random subsets from the 10,000 species-level birth–death tip-dated completed trees for 5911 species of mammal (Upham et al. 2019). For each realm, an ultrametrized consensus tree was constructed from the 1000 random trees. This was done using the “consensus.edge” and the “force.ultrametric” (with option method = ”extend”) functions of the package “phytools” (Revell 2012) implemented in the R Statistical Environment (R Core Team 2025). The polytomies were resolved using the “fix.poly” function of the R package “RRphylo” (Castiglione et al. 2018).

We used the most comprehensive-to-date molecular phylogenetic tree for fleas of Zhu et al. (2015) as the backbone for constructing realm-specific flea phylogenetic trees. Zhu et al.’s (2015) tree included the majority of flea genera, albeit not species, from our datasets. Consequently, we established the topology of the genera and species in our datasets that were not in Zhu et al.’s (2015) tree based on either their morphologically derived taxonomic positions (Hadfield et al. 2014) or the molecular and/or morphological phylogenies of several clades (see references in Krasnov et al. 2022b). All branches were assigned a length of 1 because no information on branch lengths was available. Then, the trees were ultrametrized as described above.

### Host and flea traits

We selected the host traits supposedly associated with the pattern of flea parasitism (see Krasnov 2008), namely adult body mass, relative brain mass, maximal longevity, habitat breadth, and geographic range size. Data on these traits, apart from geographic range size, were taken from the COMBINE database (Soria et al. 2021). Geographic range sizes were calculated from range maps (IUCN 2025).

We characterized each flea species by six traits, including the total number of host species exploited by the flea across its geographic range, the phylogenetic diversity of these hosts, the latitudinal span of the flea’s geographic range, its microhabitat preference (the relative time spent either in the hair of the host(s) or in its/their nest(s)/burrow(s), ranked body size (small, medium, or large), and the occurrence and number of sclerotized combs (no combs, one comb, or two combs). These data were taken from our earlier study (Krasnov et al. 2024b) (see also the rationale for flea trait selection, details of calculations, and information sources elsewhere (Krasnov et al. 2015, 2016, 2024b).

Data on quantitative traits for both hosts and fleas were standardized to have a mean of zero and a standard deviation of unity. Then, for each biogeographic realm and for hosts and fleas separately, we constructed the distance trait matrices for fleas or hosts from these data using the Gower distance coefficient with the “gowdis” function of the R package “FD” (Laliberté and Legendre 2010).

### Network structure

For each region, an interaction network was represented by a binary interaction matrix with flea species in columns, host species in rows, and cell values of 1 if a given flea species was recorded on a given host species and 0 if not. We characterized each network by two structural patterns, nestedness and modularity (see above). We calculated nestedness as the NODF index (nestedness based on paired overlap and decreasing fill; Almeida-Neto et al. 2008) that varies from 0 (non-nested network) to 100 (perfect nestedness). Modularity was calculated as Barber’s *Q* index (Barber 2007), optimized by the DIRTLPAwb + algorithm (Beckett 2016) that varies from 0 (no modules) to 1 (completely separated modules). Both metrics were computed using the “networklevel” function of the R package “bipartite” (Dormann et al. 2008, 2009). We standardized both indices by using null models (using the “shuffle.web” function of the “bipartite” package and constructing 1000 reshuffled matrices) and Z-scores. This was done (a) because regional networks differed substantially in the numbers of host species, parasite species, and interactions and (b) because network structure (both nestedness and modularity) often correlates with species richness (Olesen et al. 2007; Fonseca et al. 2005).

### Data analyses

We used two methods aimed at understanding the environmental, host-related, and flea-related drivers of the flea-mammal network structure, separately for each of the four biogeographic realms. First, we applied generalized linear models (GLM) with the response variable being either nestedness or modularity values (Z-scores; see above) and the predictors being (a) environmental and geographic variables (the principal components of temperature, precipitation, and seasonality variables, net primary production, and the absolute value of latitude), (b) host-associated variables (species richness and phylogenetic and functional diversity), and (c) flea-associated variables (species richness and phylogenetic and functional diversity). The phylogenetic and functional diversities of hosts or fleas were calculated with the “treeUniqueness” and “uniqueness” functions, respectively, of the R package “adiv” (Pavoine 2020, 2024). Prior to analyses, all response and explanatory variables were standardized as described above. For each response variable, we ran GLMs with all possible combinations of response variables and then selected the best model based on the Akaike Information Criterion (AIC) using the “model.sel” function from the R package “MuMIn” (Bartoń 2024).

Second, we tested the associations between two response matrices of network dissimilarity (see below) and seven explanatory distance matrices (environmental dissimilarity, dissimilarities in host and flea species composition, host and flea phylogenetic dissimilarities, and host and flea functional dissimilarities). This was done separately for each biogeographic realm. To estimate pairwise dissimilarity between regional networks, we used a dissimilarity metric, the *D* statistic, based on graph theory (Schieber et al. 2017). This metric quantifies dissimilarity in the network topological structure without taking into account the interactors’ species identities, answering the question of whether two graphs are identical. In other words, the *D* statistic estimates the dissimilarity of two networks from the purely structural perspective. It considers three aspects of between-network differences, namely (a) dissimilarity in average node connectivity, (b) dissimilarity in the node dispersion metric (i.e., the distributions of distances between nodes), and (c) dissimilarity in node centrality; it is calculated as the sum of these three terms, with weights set to scale each of the three components’ relative importance [see Schieber et al. (2017) and Dallas and Poisot (2018)]. Schieber et al. (2017), based on the empirical results, recommended setting these weights as 0.45, 0.45, and 0.05, respectively. The *D* statistic, therefore, mainly reflects dissimilarity in node connectivity and node distance distributions. Another advantage of the *D* statistic is that it can be applied to comparisons of differently sized networks because its calculation involves differences in the network diameters (Schieber et al. 2017). The *D* statistic ranges from 0 to 1 and has recently been applied for estimating dissimilarity between host-parasite networks (Dallas and Poisot 2018; Krasnov et al. 2024a). The *D* statistic was calculated following Dallas and Poisot (2018), using modification of their R code (see Krasnov et al. 2024a). This was done by splitting a regional bipartite host-flea network into two unipartite components, namely (a) a network of flea species connected by shared host species (host-sharing-among-fleas network; *Dh*) and (b) a network of host species connected by shared fleas (flea-sharing-among-hosts network; *Df)*. Host-sharing-among-fleas networks provide information on hosts in between-flea links, whereas flea-sharing-among-hosts networks provide information on fleas in between-host links (Dallas and Poisot 2018).

Environmental distances were calculated as Euclidean distances based on eight climatic and environmental variables (see above). Compositional dissimilarities for either fleas or hosts were calculated using the Bray–Curtis index implemented in the “vegdist” function of the R package “vegan” (Oksanen et al. 2022). Phylogenetic and functional dissimilarity matrices were constructed using the “evodiss_family” and “PADdis” functions, respectively, of the “adiv” package. For each biogeographic realm, the association between structural dissimilarity *Dh* or *Df* and the predictor distance matrices was tested using multiple regression on distance matrices (MRM; Legendre et al. 1994), using the “MRM4” function, which is based on the “MRM” function of the R package “ecodist” (Goslee & Urban 2007) and modified by Dambros et al. (2020). Prior to running the MRM, we standardized the dissimilarity values in each matrix to zero mean and unit variance using the “decostandDist” function of Dambros et al. (2020). The unique and shared effects of the predictor matrices (environmental dissimilarity and combined compositional, phylogenetic, and functional dissimilarity between hosts or between fleas) were estimated using variance partitioning analyses carried out with the “varpart4” function, based on the “varpart” function of the “vegan” package and modified by Dambros et al. (2020).

## Results

**Fig. 1:**
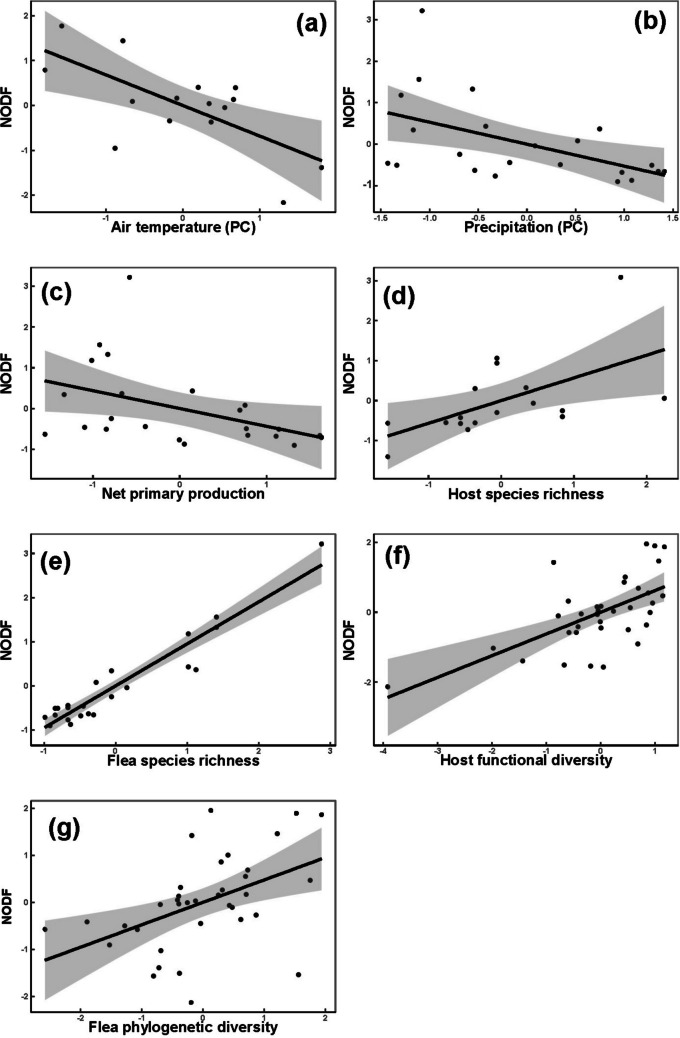
Relationships between the values of nestedness (NODF) and air temperature (principal component) in the Afrotropics (**a**); precipitation (principal component) (**b**) and net primary production (**c**) in the Nearctic; host species richness in the Neotropics (**d**); flea species richness in the Nearctic (**e**); and host functional (**f**) and flea phylogenetic (**g**) diversity in the Palearctic

**Fig. 2:**
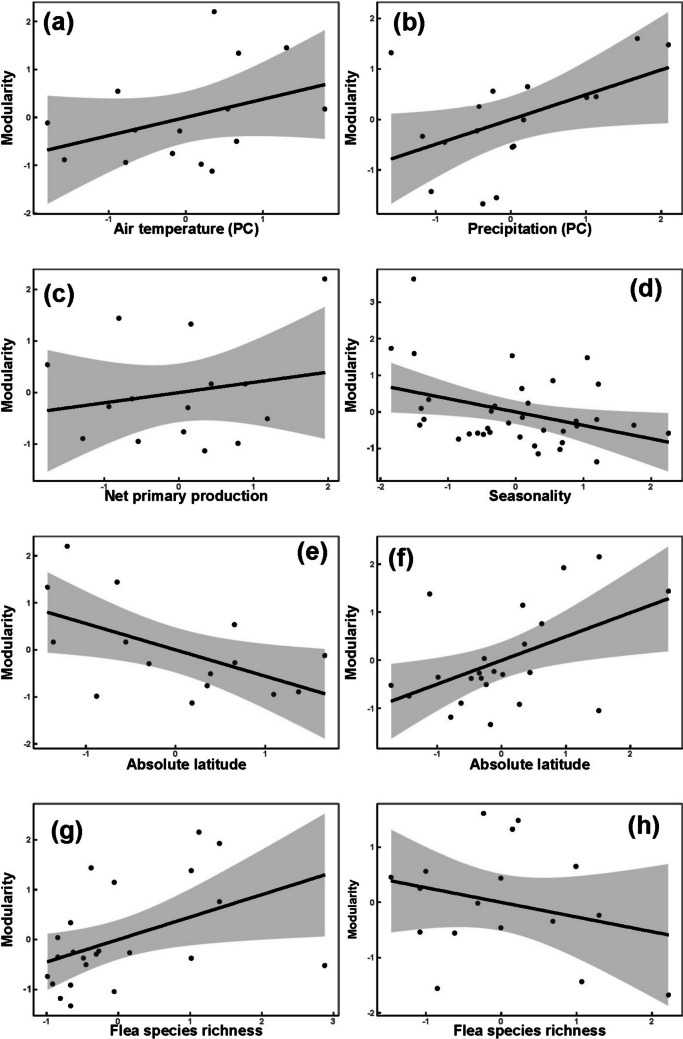
Relationships between the values of modularity and air temperature (principal component) in the Palearctic (**a**); precipitation (principal component) in the Neotropics (**b**); net primary production in the Afrotropics (**c**); seasonality (principal components) in the Palearctic (**d**); absolute latitude in the Afrotropics (**e**) and the Nearctic (**f**); and flea species richness in the Palearctic (**g**) and the Neotropics (**h**)

The best models of the effects of environmental, geographic, and interactor-associated variables produced by the GLM analyses are presented in Table 1. The effect of air temperature on nestedness was detected in the Afrotropics and the Neotropics only and on modularity in the Palearctic only, with nestedness decreasing at higher temperatures, whereas modularity increased (Figs. 1a and 2b, respectively). Precipitation correlated negatively with nestedness in the Nearctic (Fig. 1b) and positively with modularity in the Afrotropics and the Neotropics (Fig. 2b). An increase in regional productivity (net primary production) was accompanied by a decrease in the network nestedness in the Nearctic (Fig. 1c) and an increase in the network modularity in the Afrotropics (Fig. 2c) and the Neotropics. A significant relationship between seasonality and network structure was found for modularity only and in the Palearctic only. Lower modularity values were characteristic for regions with more pronounced seasonality (Fig. 2d). In contrast to the NODF values, modularity values varied with latitude in three of four realms (except the Palearctic). In all these realms, higher modularity values were found in the networks in regions farther from the Equator (see examples for the Afrotropics and the Nearctic in Fig. 2e and f, respectively). Greater NODF values were associated with greater network host species richness in three realms (except the Palearctic; see example for the Neotropics in Fig. 1d), whereas flea species richness demonstrated this effect in the Neotropics only (Fig. 1e). The values of modularity were not affected by host species richness in any realm, whereas the opposite was true for flea species richness in the Nearctic, Palearctic, and Neotropics, with this effect being positive in the two former realms (Fig. 2g for the Palearctic) and negative in the latter realm (Fig. 2h). A correlation between the network structural features and the functional or phylogenetic diversity of interactors was detected for nestedness, but not for modularity, and in the Palearctic only. The values of nestedness increased in the networks with more functionally diverse hosts and/or more phylogenetically diverse fleas (Fig. 1f and g, respectively).

**Table 1:** Summary of the best models of the effects of environmental, geographic, and host- and flea-associated variables on nestedness (NODF) and modularity (Q) (*Z*-scores) for regional host-flea networks in four biogeographic realms

Realm	NODF/Q	Equation, NODF = or Q =	*R*^*2*^	*F*	*p*
Afrotropics	NODF	-0.71*T + 0.42*HSR	0.64	10.47	0.002
	Q	2.11*P—0.69*Lat + 2.11*NPP	0.62	5.91	0.01
Nearctic	NODF	-0.29*P—0.21*NPP + 0.44*HSR + 0.49*FSR	0.95	85.35	< 0.001
	Q	0.50*Lat + 0.46*FSR	0.45	8.34	0.002
Neotropics	NODF	-0.52 *T + 0.56*HSR	0.59	10.20	0.002
	Q	2.11*P—2.49*NPP—0.56*Lat—0.54*FSR	0.70	6.98	0.004
Palearctic	NODF	0.28*FP + 0.52*HF	0.46	13.80	< 0.001
	Q	0.35*T + 1.25*P—0.54*S—1.43*NPP + 0.63*FSR	0.59	8.68	< 0.001

*T, P, and S* principal components of air temperature, precipitation, and seasonality raw variables, respectively (see text for explanation); *NPP* net primary production; *Lat* absolute latitude; *HSR and FSR* host and flea, respectively, species richness; *HP and FP*: host and flea, respectively, phylogenetic diversity; *HF* host functional diversity (no significant effect of flea functional diversity was found). Only significant coefficients are shown

Multiple regression on distance matrices demonstrated that environmental dissimilarity predicted dissimilarity in the structure of host-sharing, but not flea-sharing, networks (Table 2). Moreover, this was the case in the biogeographic realms of the Old, but not the New, World, with the Palearctic networks situated in similar environments being more structurally similar. However, the opposite trend was detected in the Afrotropics where structurally similar networks occurred in different environments (Table 2). One to three host-associated dissimilarity matrices predicted the structural dissimilarity of host-sharing-by-fleas network dissimilarity in all realms. Host compositional and/or phylogenetic dissimilarity were good predictors of this structural dissimilarity in three of the four realms, whereas host functional dissimilarity predicted host-sharing-by-fleas networks dissimilarity in the Nearctic only. In addition, the effect of flea-associated dissimilarity matrices on the structural dissimilarity of host-sharing-by-fleas networks was detected in the New, but not the Old, World realms (Table 2). The structural dissimilarity of flea-sharing-by-hosts networks was affected by flea-associated dissimilarity in the Afrotropics (phylogenetic dissimilarity) and the Nearctic (functional dissimilarity) (Table 2). Patterns of the effect of flea-associated dissimilarity on the dissimilarity of flea-sharing-by-hosts networks in these two realms were opposite.

**Table 2:** Regression coefficients and pseudo *p*-values from the permutation tests (Legendre et al. 1994) of multiple regression on distance matrices (MRM) relating dissimilarity in the network structure (host-sharing dissimilarity *Dh* and flea-sharing dissimilarity *Df*; calculated using the *D* statistic, see text for explanations) to environmental dissimilarity (dE), dissimilarity in host or flea species composition (dHC and dFC, respectively), host or flea phylogenetic dissimilarity (dHP and dFP, respectively), and host or flea functional dissimilarity (dHF and dFF, respectively) in four biogeographic realms

RM	EM	Afrotropics	Nearctic	Neotropics	Palearctic
*Dh*	dE	-0.48*	0.001	-0.12	0.22*
	dHC	1.23*	-0.04*	-0.33	-0.29*
	dHP	-0.34	0.01*	0.64*	0.25*
	dHF	0.50	-0.06*	-0.24	-0.04
	dFC	-0.09	-0.01	-0.92*	-0.14
	dFP	0.06	0.001	0.50	0.13
	dFF	0.02	-0.01*	-0.48*	0.11
*Df*	dE	0.05	0.03	-0.04	0.14
	dHC	0.52	0.17	-0.05	0.002
	dHP	0.29	-0.09	-0.03	0.04
	dHF	0.34	-0.04	-0.12	0.06
	dFX	0.13	-0.10	0.53	-0.06
	dFP	-0.57*	0.10	-0.28	-0.09
	dFF	0.25	0.18*	0.34	0.003

Asterisks denote significant coefficients. *RM* response matrix; *EM* explanatory matrix

**Fig. 3:**
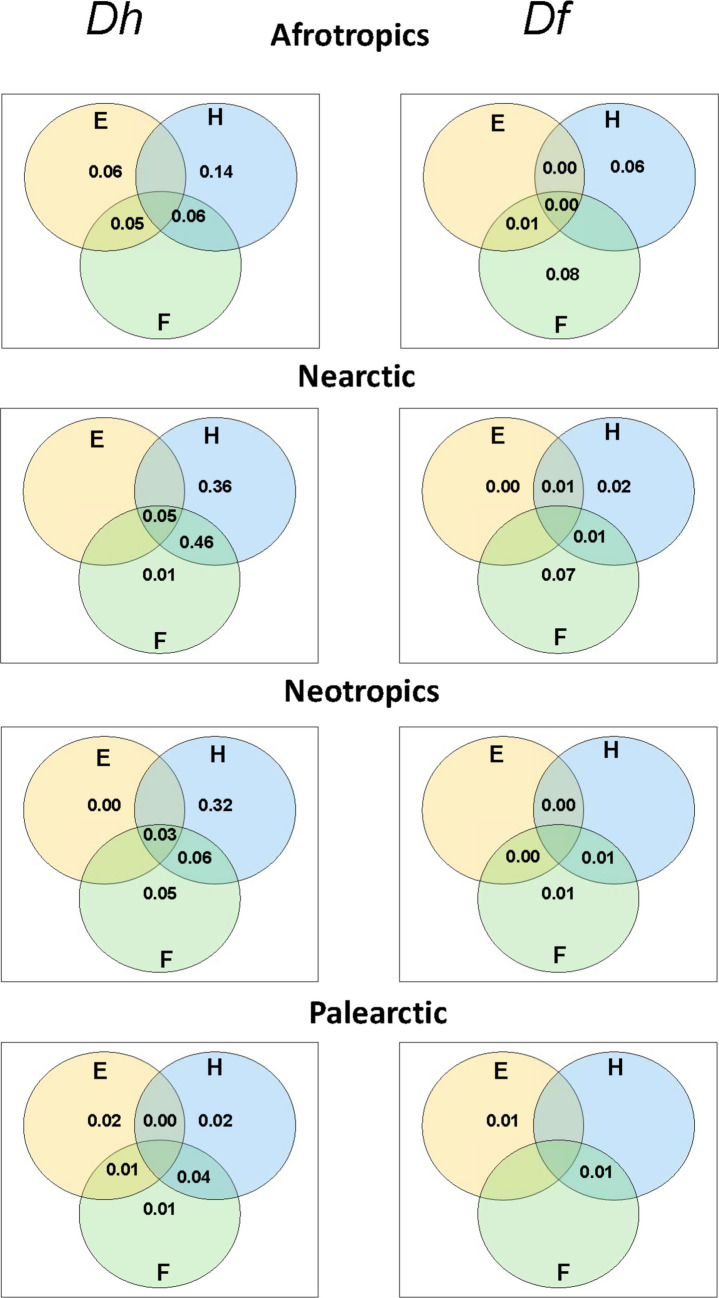
Venn diagrams demonstrating the relative contributions of three groups of explanatory distance matrices (E: environmental dissimilarity, H: combined dissimilarity in host species richness, host phylogenetic dissimilarity, and host functional dissimilarity, F: combined dissimilarity in flea species richness, flea phylogenetic dissimilarity, and flea functional dissimilarity) to explaining the variation in dissimilarity in host-flea network structure (host-sharing dissimilarity *Dh* and flea-sharing dissimilarity *Df*). Negative values not shown

When the variance in the dissimilarities of host-sharing networks was partitioned among the predictor distance matrices in the MRM, host-associated dissimilarity was the factor with the highest unique contribution, explaining the variation in this aspect of the structural dissimilarity of host-sharing-by-fleas (Fig. 3). This, however, was not the case for the Palearctic, where the largest (albeit having rather low explanatory power) part of the variation was explained by the combined effect of host- and flea-associated differences (Fig. 3). The highest unique contribution explaining the variance in the dissimilarity of flea-sharing-by-hosts networks in the Afrotropics and the Nearctic was that of flea-associated differences, whereas no predominant factor affecting the changes in the dissimilarities of flea-sharing-by-hosts networks was found for the Neotropical or the Palearctic networks (Fig. 3).

## Discussion

We found that the drivers of nestedness and modularity in host-flea networks differed between biogeographic realms. This was demonstrated by the results of both types of analyses. Although both nestedness and modularity were affected by environmental and interactor-associated factors, networks in different realms responded to different factors. In addition, the directions of some of these effects (absolute latitude and flea species richness on modularity and environmental dissimilarity and host compositional dissimilarity on *Dh*) were opposite in different realms. Among interactor-associated factors, host species richness was most often detected as an important driver of nestedness, whereas flea species richness mainly affected modularity. The dissimilarity of host-sharing-by-fleas networks was mostly explained by host-associated dissimilarity, whereas the effects of flea-associated dissimilarity on the dissimilarity of either host-sharing-by-fleas or flea-sharing-by-hosts networks were weak (if at all). No effect of host-associated dissimilarity on the dissimilarity of flea-sharing-by-hosts networks was detected. Environmental dissimilarity weakly affected *Dh* and did not affect *Df.*

Empirical studies of the effects of environmental factors and their stability on the network structure produced contradictory results. For example, the relationships between nestedness and temperature fluctuations were found to be either positive (Sebastián-González et al. 2015; Song et al. 2017; Corro et al. 2022) or negative (Welti and Joern 2015). Similarly, the modularity of bird frugivory networks decreased with an increase in annual temperature (Schleuning et al. 2014), whereas the opposite was found for bat frugivory networks (Corro et al. 2022). The discrepancies in the patterns of variation in nestedness and modularity, along environmental gradients, are likely related to differences in the networks’ ecological types (e.g., Welti and Joern 2015). Indeed, a recent meta-analysis involving 723 bipartite networks of plant-pollinator, seed-dispersal, plant-ant, plant-herbivore, and host-parasite interactions did not find a strong effect of seasonality on structural network metrics, including nestedness and modularity (Brimacombe et al. 2022). Although Brimacombe et al. (2022) explained the lack of strong general patterns by differences in sampling and network construction methodologies, they, nevertheless, recognized that it may have been related to differences between networks in their inherent biological properties (e.g., Mello et al. 2011). Variation in network structure along environmental gradients undoubtedly arises from variation in the species compositions of both consumers and resources and their ecological characteristics. In particular, variation in both the nestedness and the modularity of host-parasite networks is likely associated with variation in the number of parasite species demonstrating certain levels of specialization (Lafferty et al. 2008; de Angeli Dutra and Poulin 2024). For example, Krasnov et al. (2005) found that the degree of the host specificity of fleas was associated with the size of their geographic range, whereas this size predictably varied with latitude, so that flea host specificity varied with latitude as well (Krasnov et al. 2008). Furthermore, variation in host specificity among populations of the same flea species was found to be affected by local environmental conditions, such as air temperature and precipitation (Krasnov et al. 2004). Another reason behind the differences in nestedness and modularity values in different environments might be the so-called interaction rewiring, when the same consumer and resource species interact in one, but not in another, environment (e.g., Carstensen et al. 2014; CaraDonna et al. 2017). Originally, the concept of interaction rewiring was most often related to temporal variation in network structure, but it has also been associated with spatial variation. Environmental effects on interaction rewiring have been shown for various bipartite networks (Simanonok and Burkle 2014; Trøjelsgaard et al. 2015; Noreika et al. 2019; Henriksen et al 2022). The effects of climatic and vegetation variables on interaction rewiring in host-flea networks have been reported by Krasnov et al. (2023), although these effects were found in European and Asian, but not in South American and African, networks. Regarding fleas, spatial/environmental variation in the interaction rewiring is most likely related to these parasites’ characteristic life histories. Pre-imaginal development in the majority of flea species takes place solely off-host, in hosts’ burrows or nests. As a result, fleas are most abundant and diverse in small burrowing mammals, such as rodents and shrews (Marshall 1980; Krasnov 2008). Both imago and pre-imaginal fleas are sensitive to air temperature and relative humidity, with the degree of this sensitivity varying among flea species (e.g., Krasnov et al. 2001). The architecture and substrates of burrows and nests, belonging to the same host species, vary in dependence on environmental factors (e.g. Shenbrot et al. 2002). As a result, the same flea species interact with the same host species in one environment (where its burrow’s microclimatic conditions are favorable for this flea) and does not interact with it in another environment (when these conditions are not suitable) but rather switch to another host species with suitable burrows, although both fleas and hosts inhabit both environments (Krasnov et al. 1998).

Host species richness was the only host-associated factor affecting network structure. Moreover, this effect was found for nestedness but not for modularity. On the contrary, flea species richness affected modularity in three of the four biogeographic realms, whereas its effect on nestedness was detected in the Palearctic only. This suggests that hosts and fleas play different roles in determining network structure. The most likely reason behind this difference is the asymmetrical host-flea relationship, in that flea species select their host species rather than vice versa. The relationship between host species richness and the nestedness of host-flea networks found in our study appeared to not be the case for other host-parasite associations. For example, the nestedness of avian-haemosporidian networks was not affected by host taxonomic diversity (de Angeli Dutra and Poulin 2024). This can be explained by the passive haemosporidian transmission between hosts, on one hand, and the active selection of hosts by fleas. However, the effect of host species richness on nestedness was not found for the Palearctic. This perhaps could be due to the fact that flea fauna in the Palearctic is much richer than that in any other biogeographic realm, resulting in the relatively higher specialization level of Palearctic fleas, meaning that each Palearctic flea interacts with fewer host species than fleas from other realms (Medvedev 2005).

Associations between the modularity of networks and flea, but not host, species richness contradicts, to some extent, earlier findings of the relative roles of hosts and fleas in the modularity of flea-mammal networks. For example, Krasnov et al. (2012) reported that host, but not flea, phylogeny affected the module composition of the Palearctic host–flea networks. However, the manifestation of a modular structure seems to be mainly affected by flea-associated factors. This suggests that resources and consumers in a bipartite network might determine different facets of the same structural feature.

Dallas and Poisot (2018) were the first to apply the *D* statistic as a metric for the pure structure of mammal-helminth networks to investigate spatial variation in network structure. They related *Dh* and *Df* to the spatial distance gradient and did not find a strong distance-decay pattern, suggesting that networks situated at different distances have similar structures. Dallas and Poisot (2018) suggested that either (a) different host and parasite species play similar functional roles in interaction networks or (b) the variation in network structural similarity may be better explained by other geographic variables. The latter suggestion seems to be more likely because we found the links between host- and flea-associated dissimilarities as well, to a lesser degree, environmental dissimilarities and network structural dissimilarity. An additional reason for the lack of the distance-decay pattern reported by Dallas and Poisot (2018) could be the nature of their data, which encompassed helminth species belonging to different orders and phyla. Helminths demonstrate an enormous variety of life cycles, modes of transmission, and relationships with their hosts, so some patterns characteristic for some helminth lineages could be masked. In contrast, relationships between small mammals and the majority of fleas are surprisingly similar, with practically no variation in their life cycles and transmission modes. Moreover, a distance-decay pattern has not been detected for the pure structural dissimilarity (measured following Dallas and Poisot 2018) of mammal-flea networks in the Afrotropics, Nearctic, or Palearctic, but was found for the Neotropical networks (Krasnov et al. 2024a, b), also suggesting the role of host and parasite species composition in the manifestation of this pattern. In this study, we found that the factors associated with structural dissimilarity differed between host-sharing-by-fleas and flea-sharing-by-hosts networks. Importantly, the dissimilarity of host-sharing-by-fleas networks was determined by host-associated and, to a lesser degree, flea-associated dissimilarities, whereas the dissimilarity of flea-sharing-by-hosts networks was affected solely by flea-associated dissimilarities, although in two of the four realms only. This suggests that host sharing was determined by the reciprocal responses of fleas to hosts and hosts to fleas, whereas flea sharing depended on the responses of fleas to hosts. Again, the reason for this could be the asymmetrical host-flea relationship, with fleas actively selecting their hosts, whereas hosts defend themselves against fleas, albeit not necessarily successfully. In addition, the weak (if at all) effect of environmental dissimilarity emphasizes the main role played by interactors in determining the network structure, even when their identities are not taken into account.

The variation in the drivers of host-flea network structure between networks from different biogeographic realms is one of the most important results of this study. Moreover, even when the network structure in different realms was found to be affected by the same factor, the directions of this effect were sometimes opposite. This supports the results of our earlier studies demonstrating differential patterns of various metrics and processes in host-flea networks between biogeographic realms (Krasnov et al. 2022a, b, 2023, 2024a, 2025). Between-realm variation in the drivers of host-flea network structure can stem from several interrelated processes. First, the environmental variability and distribution of different environments are manifested differently in different realms. Second, host and flea species composition differ between realms because of (a) the differential evolutionary histories of both hosts and fleas in different realms and (b) the historical patterns of host dispersal followed by flea dispersal due to plate tectonics and/or environmental changes (Traub 1985; Medvedev 2005; Zhu et al. 2015). This presumably could lead to between-realm differences in (a) species-specific host and flea responses to these environmental factors and environmental variability and (b) the differential role of environment in mediating host-flea relationships (see Krasnov 2008 for review).

In conclusion, between-realm differences in the drivers of network structure could result from an interplay of ecological and historical factors. However, independently of between-realm differences in host and flea responses to environmental factors and to each other, the asymmetry in host-flea relationships holds everywhere. This is the most likely reason behind the differences between host-associated and flea-associated factors in their effects on the interaction network structure.

## Data Availability

The data used in this study can be found in Mendeley Data repository (Krasnov et al. 2025).
